# Complete Genome Sequence of *Bacillus thuringiensis* subsp. *jinghongiensis* Reference Strain YGd22-03

**DOI:** 10.1128/genomeA.00740-17

**Published:** 2017-09-28

**Authors:** Yan Wu, Yajuan Fu, Yihui Yuan, Meiying Gao

**Affiliations:** a Wuhan Institute of Virology, Chinese Academy of Sciences, Wuhan, China; b University of Chinese Academy of Sciences, Huairou, Beijing, China

## Abstract

*Bacillus thuringiensis* is widely used in producing ecofriendly microbial agents for the purpose of controlling insect pests. In this study, we determined the complete genome sequence of *B. thuringiensis* subsp. *jinghongiensis* reference strain YGd22-03, which contains three *cry* genes and one cerecidin biosynthetic gene cluster.

## GENOME ANNOUNCEMENT

*Bacillus thuringiensis* is an ecofriendly biopesticide for controlling a wide variety of insect pests of several orders, such as *Lepidoptera*, *Diptera*, *Coleoptera*, *Hemiptera*, *Hymenoptera*, and *Mallophaga* (1). Until now, *B. thuringiensis* strains have been classified by their flagellar immunological reactions into at least 71 H serotypes containing 84 subspecies (2, 3). YGd22-03 is a novel *B. thuringiensis* strain isolated from China and was identified as the reference strain of *B. thuringiensis* subsp. *jinghongiensis* (serotype H42) (4). The complete genome sequence of YGd22-03 was determined in this study.

Genomic DNA of YGd22-03 was extracted and a 500-bp paired-end insert library was sequenced on an Illumina HiSeq 2500 platform with read lengths of 150 bp. The reads were quality filtered by Quake (5), resulting in a 2.169-Gbp total length that comprised 17,358,564 high-quality reads and represented 400-fold coverage of the genome. The clean reads were then *de novo* assembled with a SPAdes 3.5.0 assembler (6), and 79 contigs longer than 500 bp were generated. The contigs were assembled by comparing them to multiple complete genome sequences of *B. thuringiensis* to obtain a draft genome with gaps. The gaps were further filled by high-fidelity PCR and Sanger sequencing to construct the complete genome. The genome was annotated using the NCBI Prokaryotic Genome Annotation Pipeline (https://www.ncbi.nlm.nih.gov/genome/annotation_prok/) with the best-placed reference protein set and GeneMarkS+ methods.

The complete genome of YGd22-03 contains one circular chromosome and five circular plasmids. The length of the chromosome is 5,420,545 bp, and it harbors 5,721 genes, including 36 rRNA genes and 99 tRNA genes. The G+C content of the chromosome is 35.2%. The five circular plasmids containing 315 protein-coding genes are named pYGD98 (98,137 bp), pYGD83 (83,894 bp), pYGD36 (36,974 bp), pYGD30 (30,343 bp), and pYGD5 (5,650 bp), and the G+C contents of the plasmids range from 32.0% to 34.5%.

Three parasporal crystal protein-coding genes (BVH75-30100, BVH75-30365, and BVH75-30370) and one vegetative insecticidal protein (VIP)-like protein-coding gene (BVH75-29980) are found in plasmid pYGD98. The gene products of BVH75-30365 and BVH75-30370 showed 100% amino acid sequence similarity to the proteins PS5-1 (Cry64Ba1) and PS5-2 (Cry64Ca1), respectively. The gene product of gene BVH75-30100 shows 99% similarity to Cry11-like protein (GenBank accession number AKS28920). According to a previous report, the gene products from one or all three of these *cry* genes might exhibit cytocidal activity against cancer cells (7). In addition, a cerecidin biosynthetic gene cluster, whose product was reported to exhibit bactericidal activity against a broad spectrum of Gram-positive pathogens (8), was found in the chromosome by analysis of the genome with antiSMASH (9). Therefore, YGd22-03 might be a candidate for potential medical usage for harboring anticancer proteins and producing the antimicrobial substance cerecidin.

### Accession number(s).

The genome sequence of the *B. thuringiensis* strain YGd22-03 has been deposited in GenBank under the accession numbers CP019230 to CP019235. The strain is available in the *Bacillus* Genetic Stock Center (http://www.bgsc.org/) under the identification number BGSCID 4AR1.
